# Hypertensive disorders in pregnancy and maternal and neonatal outcomes in Haiti: the importance of surveillance and data collection

**DOI:** 10.1186/s12884-019-2361-0

**Published:** 2019-06-20

**Authors:** Matthew Bridwell, Endang Handzel, Michelle Hynes, Reginald Jean-Louis, David Fitter, Carol Hogue, Reynold Grand-Pierre, Hedwige Pierre, Bradley Pearce

**Affiliations:** 1 U.S. Centers for Disease Control and Prevention, Atlanta, GA USA; 2 Department of Epidemiology, Rollins School of Public Health, Emory University, Atlanta, GA USA; 3 Ministère de la Santé Publique et de la Population, Port-au-Prince, Haiti; 4 Hôpital Albert Schweitzer, Decapelle, Haiti

**Keywords:** Hypertensive disorders in pregnancy, Hypertension, Preeclampsia, Eclampsia, Pregnancy, Stillbirth, Haiti

## Abstract

**Background:**

This study aims to determine reported prevalence of hypertensive disorders in pregnancy (HDP) and maternal and neonatal outcomes associated with these disorders among women delivering at selected hospitals across Haiti.

**Methods:**

A retrospective review of 8822 singleton deliveries between January 2012 and December 2014 was conducted at four hospitals in separate Departments across Haiti. Researchers examined the proportion of women with reported HDP (hypertension, preeclampsia, eclampsia) and the association between women with HDP and three neonatal outcomes: low birth weight, preterm birth, and stillbirths; and two maternal outcomes: placental abruption and maternal death in Hôpital Albert Schweitzer (HAS). Odds ratios for associations between HDP and perinatal outcomes at HAS were assessed using logistic regression, adjusting for potential confounders.

**Results:**

Of the 8822 singleton births included in the study, 510 (5.8%) had a reported HDP (including 285 (55.9%) preeclampsia, 119 (23.3%) eclampsia, and 106 (20.8%) hypertension). Prevalence of HDP among each hospital was: HAS (13.5%), Hôpital Immaculée Conception des Cayes (HIC) (3.2%), Fort Liberté (4.3%), and Hôpital Sacré Coeur de Milot (HSC) (3.0%). Among women at HAS with HDP, the adjusted odds of having a low birth weight baby was four times that of women without HDP (aOR 4.17, 95% CI 3.19–5.45), more than three times that for stillbirths (aOR 3.51, 95% CI 2.43–5.06), and five times as likely to result in maternal death (aOR 5.13, 95% CI 1.53–17.25). Among the three types of HDP, eclampsia was associated with the greatest odds of adverse events with five times the odds of having a low birth weight baby (aOR 5.00, 95% CI 2.84–8.79), six times the odds for stillbirths (aOR 6.34, 95% CI 3.40–11.82), and more than twelve times as likely to result in maternal death (aOR 12.70, 95% CI 2.33–69.31).

**Conclusions:**

A high prevalence of HDP was found among a cohort of Haitian mothers. HDP was associated with higher rates of adverse maternal and neonatal outcomes in HAS, which is comparable to studies of HDP conducted in high-income countries.

## Background

Haiti is known to have one of the highest maternal mortality ratios (MMR) in the world at an estimated 359 per 100,000 live births [1]. The stillbirth rate is also high with an annual estimated rate of 15.5 stillbirths per 1000 live births, resulting in 4300 stillbirths per year [2]. Hypertensive disorders in pregnancy (HDP) are one of the leading causes of maternal mortality and stillbirths [3]. HDP includes gestational hypertension, chronic hypertension, preeclampsia, and eclampsia. Hypertension (both chronic and during pregnancy) has been identified as a significant cause of morbidity among Haitian women, yet the etiology behind this association is currently unknown [4, 5, 6, 7]. Few studies have attempted to explore hypertensive disorders among pregnant women in Haiti; thus, the true impact of HDP on Haitian mothers and babies is largely unknown. Low and middle-income countries (LMIC) are known to be disproportionally affected by HDP, yet good quality information on prevalence of HDP in these settings are still missing in the literature.

In 2012, the United States Centers for Disease Control and Prevention (CDC) partnered with the Haitian Ministry of Public Health and Population (MSPP) to set up an Enhanced Routine Pregnancy Outcome Surveillance System (ePOSS) in Haiti. The goal of this surveillance system is to improve the collection and analysis of routine epidemiologic indicators used to measure pregnancy outcomes at the facility and community levels in selected regions of Haiti [8]. ePOSS was initially established in three departments, Artibonite, Nord, and Nord-Est, which serve 11 communes collectively. The facilities that were targeted in these communes were tertiary healthcare facilities and/or comprehensive emergency obstetric care facilities (CEmOC).

**Fig. 1:**
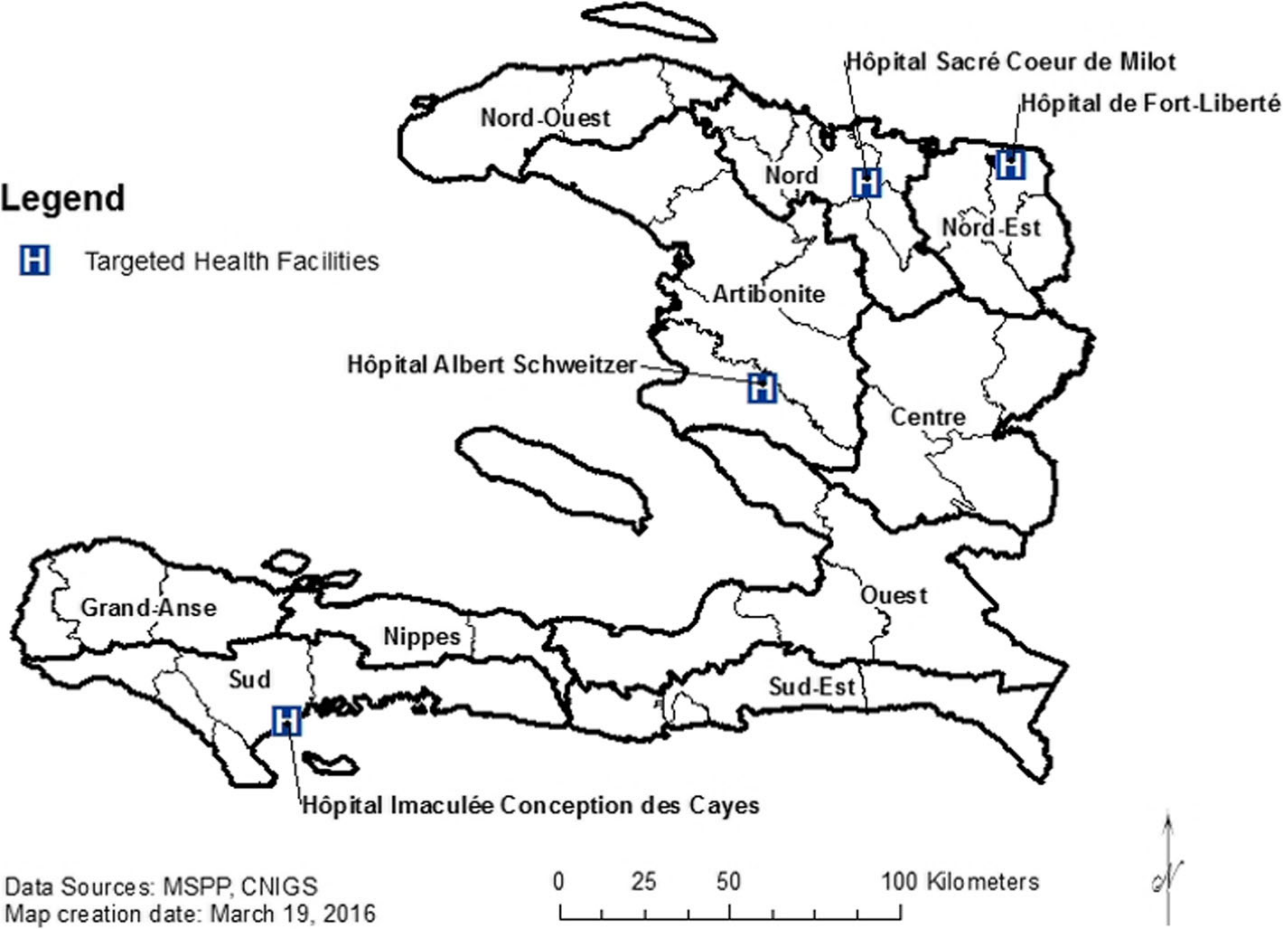
Geographic Distribution of Targeted Hospitals (ePOSS, 2012–2014)

As of October 2014, the ePOSS system had been introduced and implemented in 19 target facilities within six administrative Departments: Artibonite, Nord, Nord-Est, Sud, Sud-Est, and Nippes. Four hospitals, each representing a different department, were included in this study: Hôpital Albert Schweitzer (HAS) in Artibonite, Hôpital de Fort Liberté in Nord-Est, Hôpital Sacré Coeur de Milot (HSC) in Nord, and Hôpital Immaculée Conception des Cayes (HIC) in Sud (Fig. 1). No data were available for hospitals in Sud Est and Nippes at the time of evaluation.

Prior studies in Haiti have found a prevalence of preeclampsia and/or eclampsia among pregnant women to be 7–18% [9, 10, 11]. One of these studies by Raghuraman et al. examined associations between HDP and adverse maternal and fetal outcomes in HAS hospital records from January 2011–December 2012 [9]. Investigators found the prevalence of preeclampsia and eclampsia to be 16.6% and an association between preeclampsia and eclampsia with maternal death and placental abruption [9]. The study was limited to data from one hospital in Haiti and was unable to compare preeclamptic and eclamptic women with normotensive women due to a lack of data collected on women who were normotensive [9].

This ongoing study will expand upon the research by Raghuraman et al. by using more recent data from four hospitals in Haiti, including HAS, and exploring differences in adverse maternal and neonatal outcomes between hypertensive and normotensive women delivering at HAS. The aims of this study are to determine: (1) the prevalence of hypertensive disorders in pregnant women with singleton births delivering at four Departmental hospitals in Haiti; and (2) the extent to which certain maternal and neonatal outcomes are associated with these disorders. Answers to these questions add to the limited research on HDP in low resource areas by studying linkages of maternal and newborn outcomes. This study also allows researchers to identify areas for further improvement at the hospital level and provide data that can aid in evidence-based intervention strategies.

## Methods

### Data

Data were retrieved from the Haiti ePOSS system, which combines data from medical registries and charts. For this study, data from four hospitals (HAS, Fort Liberté, HSC, and HIC) were selected based on the following criteria: 1) being the largest referral hospitals in their distinct Departments, and 2) having the most complete records known by the research team, who participate in ePOSS. HAS and Fort Liberté had complete data from 2013 and 2014. For HSC, only 2012 data were available at the time of the analysis. For HIC, data from 2014 (its first year in ePOSS) were included.

These four hospitals are located in different Departments throughout Haiti (Fig. 1). HAS is located in Verrettes commune in Artibonite. Fort Liberté is located in the Fort Liberté commune in Nord Est; HSC is in the Milot commune in Nord; and HIC is in the Port-de-paix commune in Sud (Fig. 1).

All hospitals recorded data into multiple reporting system registries, including: maternity, major and minor operating theatre, emergency unit, and abortion (induced or late spontaneous). ePOSS is able to combine these multiple registries into a single registry for all hospitals except for HIC. Due to registry system issues, HIC still had two separate registries in ePOSS: one maternal registry and one operating theater (OT) registry. Women in the OT registry were those who required surgery, while the maternity registry captured vaginal births or low-risk cesarean sections. Both registries were used for the HDP prevalence calculation, but due to lack of data on outcomes of interest (infant status, maternal status, and birth weight) in the OT registry, only the maternal registry was used in further analyses.

The primary outcomes of interest in ePOSS are outcomes of pregnancy including maternal deaths, stillbirths, neonatal deaths, birth complications, birth defects, and live births [8]. Maternal and neonatal deaths were defined by status at discharge from hospital. An offspring’s status at birth was recorded as live birth or stillbirth (macerated, fresh, or undetermined stillbirth). Maternal, neonatal, and birth complications were recorded in three columns. Up to three complications were recorded for each birth [8].

### Sample

**Fig. 2:**
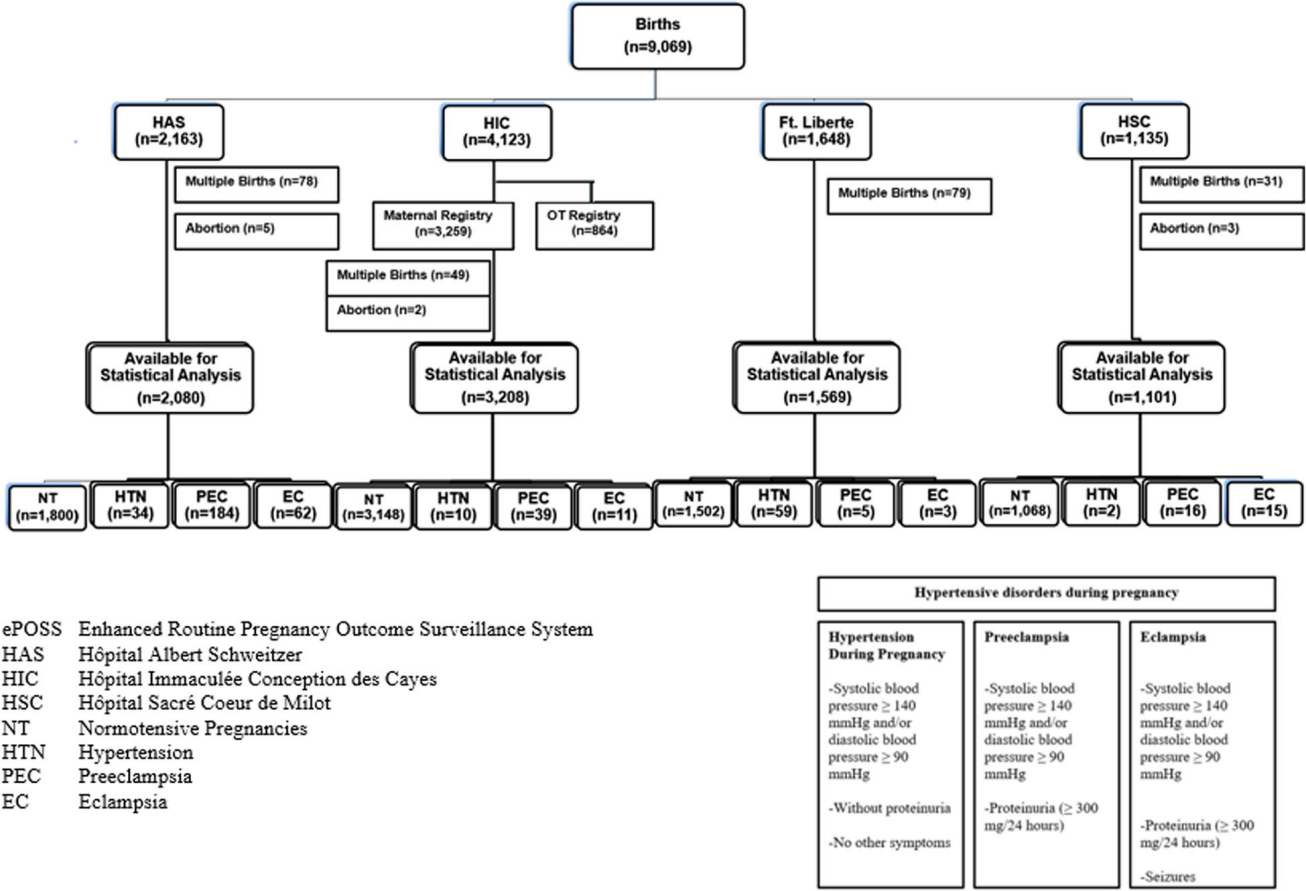
Cohort chart (ePOSS, 2012–2014)

A retrospective review was conducted of 9069 women entered into ePOSS (Fig. 2). Women who had multiple-birth pregnancies (*n* = 237) or who had an induced or late spontaneous abortion (*n* = 10) were excluded from this study. There were three cohorts used in the analyses. Prevalence was estimated using the full cohort of 8822 women. Due to a lack of further data collected at HIC’s OT Registry, 864 women had to be omitted from further analyses; thus, the second cohort size was 7958 women. The final cohort that analyses were run on included women from HAS only (*n* = 2080) (Fig. 2).

### Exposure variable

The exposure of interest was HDP, defined as a diagnosis of one the following complications during pregnancy: hypertension during pregnancy (systolic blood pressure (SBP) level ≥ 140 mmHg or diastolic blood pressure (DBP) ≥ 90 mmHg on at least two occasions, four or more hours apart after 20 weeks gestation), preeclampsia, and/or eclampsia. Preeclampsia was characterized as a SBP level of ≥140 mmHg or DBP ≥ 90 mmHg (on at least two occasions, four or more hours apart) with proteinuria (≥ 300 mg/24 h) in at least 2 urine specimens collected 6 hours or more apart. Eclampsia was defined as a SBP level of ≥140 mmHg or DBP ≥ 90 mmHg, proteinuria, and seizures [12, 13].

### Outcome variables

The variables included in this study were maternal and neonatal outcomes that are thought to be associated with HDP based on current research in high-income countries [14, 15]. The main neonatal outcomes of interest were low birth weight (< 2500 g), preterm birth (< 37 weeks’ gestation), and stillbirths (death at or after 28 weeks’ gestation). The two maternal outcomes of interest were placental abruption and maternal death (prior to hospital discharge). Other variables included in the analyses were maternal age, parity (dichotomized as nulliparous vs. parous), mode of delivery (vaginal vs. Caesarean section), and gestational age in weeks.

### Data analysis

Comparative analyses, including prevalence, between women with HDP and those without were conducted between the hospitals. Continuous data were summarized using mean and standard deviation and categorical data were presented as frequencies and percentages. Comparisons were assessed using T-tests for continuous variables, chi-square tests for categorical variables, and Fisher’s exact tests for categorical variables with small values. All significant thresholds were set at a *p*-value<0.05.

Logistic regression was used to calculate adjusted odds ratios with 95% confidence intervals using HAS data exclusively (2080 pregnancies). The outcomes analyzed were low birth weight, stillbirth, and maternal death. There were not enough cases of placental abruption to analyze further. Odds ratios were adjusted for the following potential confounders: maternal age, parity, and year of delivery (to account for time of data recording).

Statistical analyses were performed using SAS 9.4 (Cary, NC).

## Results

### HDP prevalence

Among the 8822 women with singleton births included in the study, 510 (5.8%) had a HDP. Prevalence of HDP among the selected sample at each hospital was as follows: HAS (13.5%), HIC (3.2%), Fort Liberté (4.3%) and HSC (3.0%). Among the 510 women with HDP, 285 (55.9%) were preeclamptic, 119 (23.3%) were eclamptic, and 106 (20.8%) had gestational hypertension (Table 1).

**Table 1:**
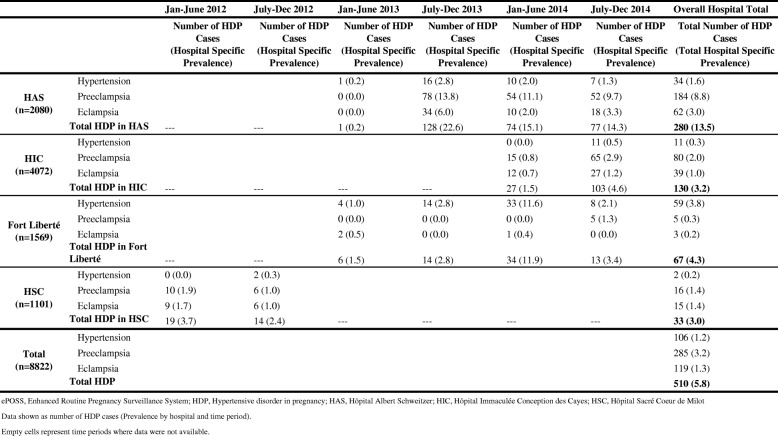
Prevalence of hypertensive disorders in pregnancy in 4 hospitals by 6 month intervals (ePOSS, 2012–2014)

### Study population characteristics and obstetric and perinatal outcomes

Of the 8822 women included in the study, clinical characteristics were available for 7958 (90.2%). These women had a mean maternal age at delivery of 27.7 years (SD = 6.7) and 4194 (52.7%) women had a prior delivery. Of the 7958 women included in the analysis, 6263 (78.7%) had a vaginal delivery. There were 738 (9.3%) preterm deliveries, 1240 (15.6%) low birth weight babies, and 392 (4.9%) stillbirths. The stillbirth rate for this cohort was 49.3 stillbirths per 1000 live births. There were 26 (0.3%) maternal deaths and 38 (0.5%) women who had a placental abruption (Table 2). The MMR in this study, classified as deaths prior to discharge, was found to be 327 deaths per 100,000 live births.

**Table 2:**
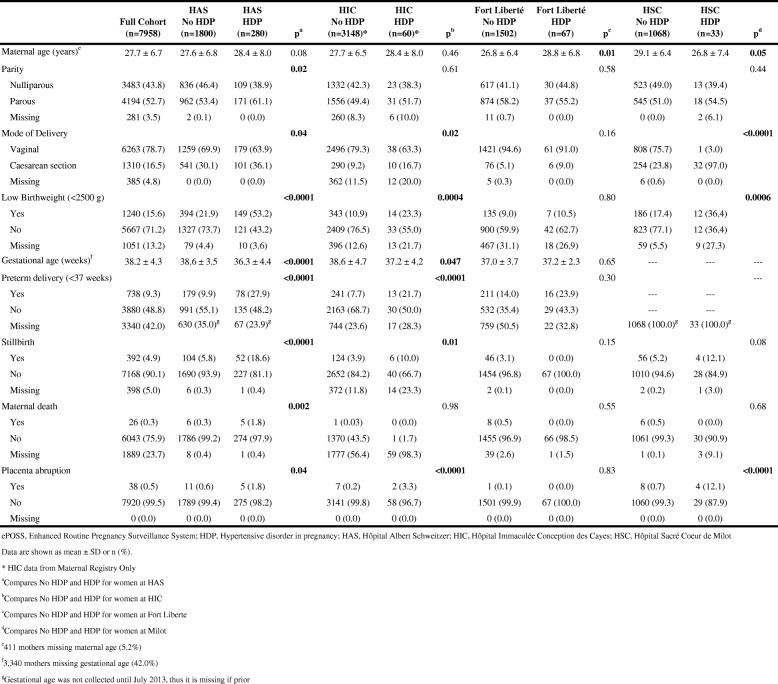
Comparison of HDP vs. No HDP and obstetric characteristics of women at 4 hospitals in Haiti (ePOSS, 2012–2014)

### Characteristics among HDP vs. no HDP associated with outcome variables

Table 2 also shows characteristics and outcomes by hospital and by HDP status. In all hospitals except Fort Liberté, women with HDP were significantly more likely to have a low birth weight baby (*p* < 0.05). Women at HAS and HIC with HDP had significantly higher proportions of preterm deliveries and stillbirths. Maternal death was significantly linked to HDP status at HAS only (*p* = 0.002), Placenta abruption was significantly more likely among women with HDP in comparison to those without HDP in all hospitals except Fort Liberté (p < 0.05).

### Characteristics among stillbirths vs. live births at HAS

There were 156 (7.5%) stillbirths and 1917 (92.2%) live births at HAS; outcome data were not available for seven (0.3%) births (Table 3). Women with stillbirths were more likely to be parous and more likely to have a vaginal birth. A stillbirth was more likely to be low birth weight. A live birth was more common among normotensive mothers, while stillbirths were more common among mothers with preeclampsia or eclampsia. A stillbirth was also significantly associated with maternal death and placenta abruptions.

**Table 3:**
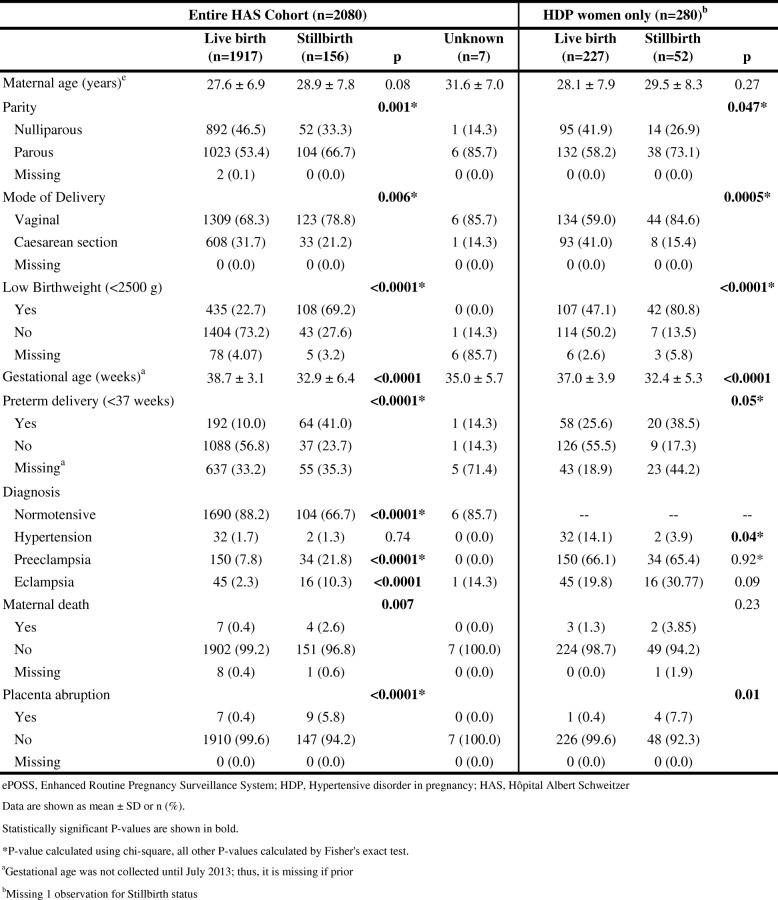
Clinical presentation of women with and without stillbirths at HAS, by entire cohort and by HDP mothers only (ePOSS, 2012–2014)

Among the 2080 women giving birth at HAS, 280 presented with HDP. Among these women with HDP, there were 52 (18.6%) stillbirths and 227 (81.1%) live births; mortality information was not available for one woman. There was no significant difference in maternal age by infant outcome. Among women with HDP at HAS, stillbirths were more likely than live births to be parous and have a vaginal delivery. More placental abruptions were seen among the women having stillbirths (Table 3).

### Characteristics among maternal deaths vs. survivors at HAS

As seen in Table 4, there were 11 maternal deaths (0.5% of women with singleton deliveries) at HAS; maternal status was not available for nine women. Women who died at HAS were more likely to have had a Caesarean section and deliver a low birth weight baby. Women who died were more likely to suffer from eclampsia and less likely to be normotensive. Those with a maternal death were also significantly more likely to deliver a stillborn baby.

**Table 4:**
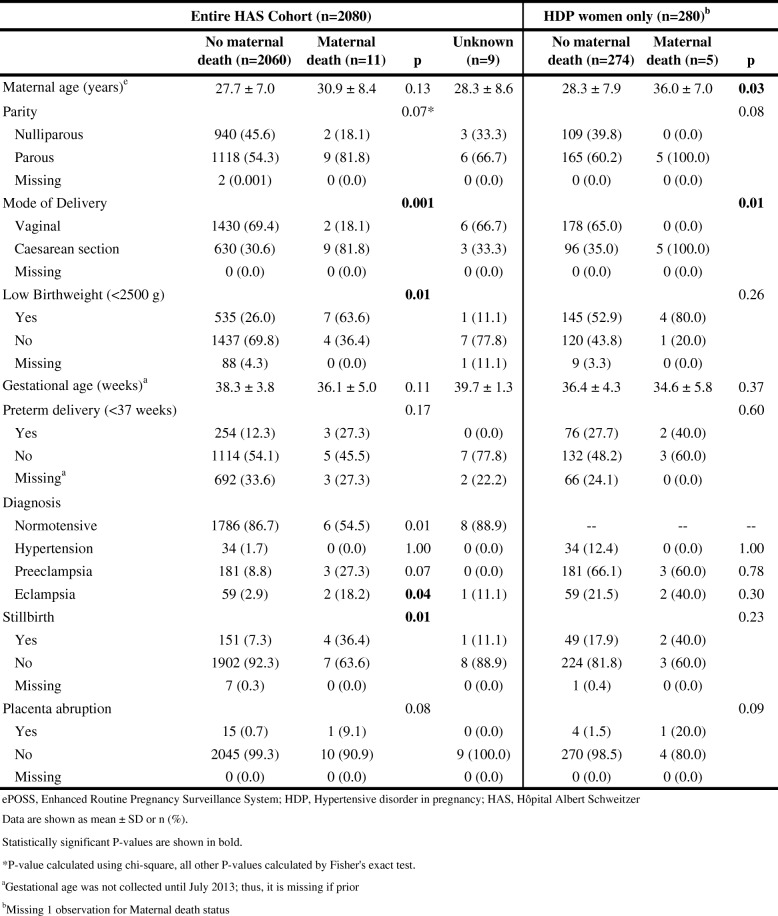
Clinical presentation of women by maternal death status at HAS, by entire cohort and by HDP women only (ePOSS, 2012–2014)

Of the 280 women at HAS with HDP, 274 mothers (97.9%) survived pregnancy and five mothers died; vital status was not available for one mother. Women with HDP who died were older (36.0 years vs. 28.3 years) and more likely to have a Caesarean section delivery. A larger proportion of these women were parous, but the difference was not statistically significant. All other adverse outcomes were not significantly different between the two groups (Table 4).

### Association between HDP and adverse outcomes

Among women at HAS who had HDP, the adjusted odds of having a low birth weight baby was four times that for women without HDP (aOR 4.17, 95% CI 3.19–5.45), more than three times for stillbirths (aOR 3.51, 95% CI 2.43–5.06), and five times for maternal death (aOR 5.13, 95% CI 1.53–17.25). Among the 3 types of HDP, eclampsia was associated with the greatest odds of adverse events with five times the odds of having a low birth weight baby (aOR 5.00, 95% CI 2.84–8.79), six times for stillbirths (aOR 6.34, 95% CI 3.40–11.82), and more than twelve times for maternal death (aOR 12.70, 95% CI 2.33–69.31). Hypertension alone was not associated with adverse outcomes. Crude odds ratios were similar to the adjusted odds ratios (Table 5).

**Table 5:**
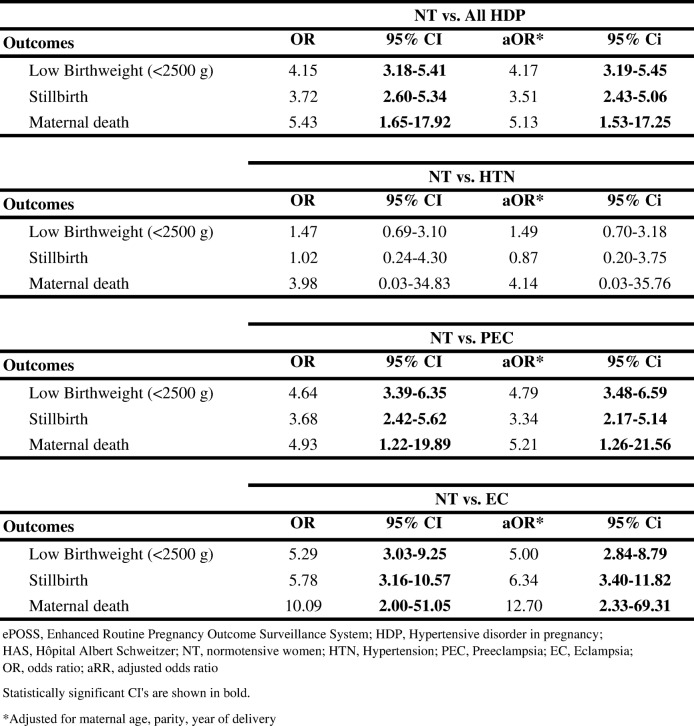
Logistic regression on the association between HDP and outcomes for women at HAS (ePOSS, 2012–2014)

## Discussion

This study examines the prevalence of HDP among Haitians, while also investigating the link between HDP and adverse pregnancy outcomes. Women with HDP in this sample were more likely to have pregnancies that resulted in low birth weight babies, stillbirths, and maternal death. Women with eclampsia were at greatest risk for these adverse maternal and neonatal outcomes.

It is estimated that HDP occur in 5–10% of all pregnancies worldwide [16]. Low and middle-income countries are known to be disproportionally affected by HDP. The few prior studies in Haiti have estimated prevalence of preeclampsia and/or eclampsia among their samples to be 7–18% [9, 10, 11]. This hospital-based study found an overall HDP prevalence to be 5.8%, with individual hospitals’ prevalence ranging from 3.0 to 13.5%. Three of the facilities (HIC, Fort Liberté, and HSC) have similar estimates, while HAS has much higher prevalence. While all 4 facilities are referral hospitals, HAS is known to be the largest referral hospital with the most established electronic medical record (EMR) system and sees the most complicated cases. It is difficult to tell whether differences in prevalence rates are due to EMR reporting and what is due to occurrence of disease in the populations presenting at the various facilities.

Haiti’s MMR is estimated to be between 359 and 389 deaths per 100,000 live births [1]. Although this study classified maternal mortality based on deaths occurring prior to discharge, a similar MMR of 327 deaths per 100,000 live births was found. The research team expects more maternal deaths would be captured if these mothers were followed for 42 days post-delivery. The lack of postpartum data is a known limitation, as a significant proportion of maternal deaths occur after women leave the hospital. Additionally, many women with complications never reach facilities, which may also lead to an underreporting of maternal mortality, HDP and other adverse outcomes.

The estimated stillbirth rate for Haiti is 15.5 stillbirths per 1000 live births [2]. The current study found a much higher stillbirth rate of 49.3 stillbirths per 1000 live births at HAS. It is likely that this hospital-based sample does not represent the true prevalence of major complications (HDP, maternal death, and stillbirth). Women without complications may be less likely to deliver at a facility, which could result in biased estimates as well.

One of the few studies that aimed to study HDP in Haiti was by Raghuraman et al. This study, while similar in topical area, included different diagnoses than the current study; thus, the prevalence in the two studies are not comparable. This study, similar to the current study, was also conducted at HAS. The previous study used data from 2011 to 2012, while the current study used data from 2013 to 2014. Researchers in the prior study obtained medical records from 1743 pregnancies, and found that 16.6% (290) of women were diagnosed with preeclampsia and eclampsia, resulting in 48 stillbirths and 5 maternal deaths [9]. The current study used records from 2080 pregnancies and found the prevalence of HDP at HAS to be 13.5% (280) with 156 stillbirths and 11 maternal deaths. Raghuraman et al. looked at women who were diagnosed with antepartum preeclampsia, antepartum eclampsia, postpartum preeclampsia, and postpartum eclampsia, while the present study analyzed diagnoses of hypertension during pregnancy, preeclampsia, and eclampsia. Chart review and systematic data entry were used to assess maternal complications for both studies, and the resulting estimates of HDP prevalence are similar. Both studies found that there was no difference in maternal age among women with HDP and who had a stillbirth compared to women who had a live birth. More maternal deaths and placental abruptions were seen among the women with HDP and who had stillbirths in both studies. Significantly more stillbirths were identified in the current study, which authors believe is due to improved surveillance at HAS and the roll-out of the electronic data base.

One key difference between this study and the Raghuraman et al. study is the ability to compare women with HDP to women without HDP in this study. The Raghuraman study lacked medical data on women with no hypertensive disorders; thus, they were unable to have a group to compare complications and other associations [9].

The current study also examined HDP in three additional hospitals in other areas of Haiti, thereby allowing comparisons among different Departments and obtaining a broader perspective on hospital-based prevalence estimates. Prevalence of HDP varied among the four hospitals, and was much higher at HAS compared to the others. This could be due to HAS having a known electronic medical records system that has been implemented for many years; thus, they may have more extensive records and better record keeping. All hospitals are in rural areas; however, HAS is the largest referral hospital in Haiti in which they receive more complicated cases from other hospitals. The lack of consistency between the four hospitals may not only be differences among samples, but also differences in surveillance and record keeping. All hospitals, except Fort Liberté, found an association between HDP and higher proportions of low birth weight babies and stillbirths. The relative small number of women with HDP at HIC, Fort Liberté, and HSC may have masked some of the associations that were seen at HAS.

The limitations to this study include the retrospective nature of this hospital-based study. These findings are not generalizable to the Haitian population due to the large number of women who do not give birth inside a hospital or medical facility. This sample represents deliveries in these four hospitals in four Departments of Haiti.

Data were collected from existing surveillance records that had incomplete records and missing data. Gestational age was not collected in the surveillance system until July 2013, which affected records from HAS, Fort Liberté, and HSC. HIC outcomes were not recorded for women presenting at the OT, which may have caused associations to be missed since women who require surgical intervention often have more high-risk pregnancies and complications. Maternal deaths at HIC were poorly reported and appear to be differentially missing; women with reported HDP appear to be missing maternal vital status more than women without HDP. Data from Fort Liberté may not be representative of the population in that commune and District as this hospital does not have a surgery ward. Women who present at Fort Liberté with a high-risk pregnancy requiring surgery are referred out to other health facilities. A further limitation to this study is the inconsistency of years of data used for the various hospitals. It is expected that more years of implementing the surveillance system may be associated with more complete and accurate data reporting.

A limitation of this study is the lack of maternal variables that were being captured in ePOSS at the time of data analysis. History of chronic hypertension and diabetes mellitus were not able to be assessed as maternal outcome variables and known complications of preeclampsia and eclampsia, such as stroke or renal failure, were not able to be assessed as potential confounders. A further limitation of this study is the lack of specific blood pressure readings. Researchers were unable to confirm the readings; thus, there is a potential for misclassification of HDP. HDP and placental abruption complications are captured in the surveillance as three complication variables. Not having a specific recording section for each of these separately leads to a potential underreporting of these data. Heavier maternal weight, which has been shown to be associated with a 3-fold odds for HDP in a community-based sample of prenatal patients in Haiti, was also not recorded in this system [11]. In that study, maternal age of more than 40 years was also found to be associated with a 3-fold odds of HDP in a multivariable model that included maternal weight and the number of prenatal visits [11].

This study adds to the limited work studying HDP in LMIC’s and in Haiti specifically. The lack of consistency between the four hospitals in this study exemplifies not only opportunities to improve surveillance in Haiti, as the true burden of HDP in this country remains unknown. Additional studies are needed to further examine the etiology of hypertension in Haitian women, especially during pregnancy. Due to the majority of Haitian mothers delivering outside of a medical facility, this large sample of the population remains unrepresented in statistics and in the literature. Qualitative and quantitative research at the community level can add to this research and allow for a comprehensive analysis of HDP among all Haitian mothers. Results from this study allows researchers to identify areas for further improvement at the hospital level and provides data that can aid in evidence-based intervention strategies. Studies like this one emphasizes the importance of detailed data collection and surveillance in order to better target investments, public health interventions, and public health policies.

## Conclusion

In conclusion, the results of this study highlight the importance of HDP as a major cause of adverse maternal and neonatal outcomes among this population in Haiti, which is comparable to studies conducted in high-income countries. Specifically, women with eclampsia, the most severe form of HDP, had the worst health outcomes including an increased odds of having a low birth weight baby, delivering a stillbirth, and were more likely to die due to complications of pregnancy. More research is needed in Haiti to understand the true prevalence and impact of HDP in facility and home births.

## Data Availability

Data and material from which this manuscript was developed are available on reasonable request from the corresponding author.

## Abbreviations

AOR: Adjusted Odds Ratio
BMI: Body Mass Index
CEmO: Comprehensive Emergency Obstetric Care Facilities
EMR: Electronic Medical Record
ePOSS: Enhanced Routine Pregnancy Outcome Surveillance System
HAS: Hôpital Albert Schweitzer
HDP: Hypertensive Disorders in Pregnancy
HIC: Hôpital Immaculée Conception des Cayes
HSC: Hôpital Sacré Coeur de Milot
LMIC: Low and middle-income countries
MDG: Millennium Development Goals
MMR: Maternal mortality ratio
NGO: Non-governmental organizations
OT: Operating Theater
SDG: Sustainable Development Goals
SOG: Soins Obstetricaux Gratuits

## References

[1] World Health Organization. Trends in maternal mortality: 1990 to 2015. 2015. Retrieved from http://apps.who.int/iris/bitstream/10665/193994/1/WHO_RHR_15.23_eng.pdf?ua=1.

[2] World Health Orgnaization; UNICEF. Stillbirths: the invisible public health problem. 2011. Retrieved from http://www.who.int/pmnch/media/news/2011/20110414_stillbirths_pressrelease.pdf.

[3] World Health Organization. Maternal mortality. 2016. Retrieved from http://www.who.int/mediacentre/factsheets/fs348/en/

[4] World Health Organization. Haiti. 2016. Retrieved from http://www.who.int/countries/hti/en/.

[5] Jiao J e a (2014). Hypertension prevalence: an examination of urban and rural Haiti. Lancet Glob Health.

[6] Dickstein Y, Neuberger A, Golus M, Schwartz E (2014). Epidemiologic profile of patients seen in primary care clinics in an urban and a rural setting in Haiti, 2010-11. Int Health.

[7] Jean-Baptiste ED, Larco P, Charles-Larco N, Vilgrain C, Simon D, Charles R (2006). Glucose intolerance and other cardiovascular risk factors in Haiti. Prevalence of diabetes and hypertension in Haiti (PREDIAH). Diabetes Metab.

[8] Handzel EW, Grand-Pierre R, Louis RJ, De Jesus S, Felker-Kantor E, et al. Pregnancy Outcome Surveillance System (POSS): Standard Operating Procedures. Centers for Disease Control and Prevention. 2014.

[9] Raghuraman N, March MI, Hacker MR, Modest AM, Wenger J, Narcisse R (2014). Adverse maternal and fetal outcomes and deaths related to preeclampsia and eclampsia in Haiti. Pregnancy Hypertension.

[10] Small MJ, Kershaw T, Frederic R, Blanc C, Neale D, Copel J, Williams KP (2005). Characteristics of preeclampsia- and eclampsia-related maternal death in rural Haiti. Journal of Maternal-Fetal & Neonatal Medicine.

[11] Sekkarie Ahlia, Raskind-Hood Cheryl, Hogue Carol (2015). The effects of maternal weight and age on pre-eclampsia and eclampsia in Haiti. The Journal of Maternal-Fetal & Neonatal Medicine.

[12] République d'Haiti Ministére de la Santé Publique et de la Population (MSPP). Protocoles de prise en charge des complications obstétricales et néonatales. 2015.

[13] World Health Organization. WHO recommendations for prevention and treatment of pre-eclampsia and eclampsia. 2011. Retreived from https://www.who.int/reproductivehealth/publications/maternal_perinatal_health/9789241548335/en/. 23741776

[14] Sibai B, Dekker G, Kupferminc M (2005). Pre-eclampsia. Lancet.

[15] Moroz Leslie A., Simpson Lynn L., Rochelson Burton (2016). Management of severe hypertension in pregnancy. Seminars in Perinatology.

[16] Cunningham FG, e. a. Williams Obstetrics (Vol. 24th edition): McGraw-hill Education; 2014.

